# The Identification and Validation of Two Heterogenous Subtypes and a Risk Signature Based on Ferroptosis in Hepatocellular Carcinoma

**DOI:** 10.3389/fonc.2021.619242

**Published:** 2021-03-02

**Authors:** Zaoqu Liu, Libo Wang, Long Liu, Taoyuan Lu, Dechao Jiao, Yuling Sun, Xinwei Han

**Affiliations:** ^1^Department of Interventional Radiology, The First Affiliated Hospital of Zhengzhou University, Zhengzhou, China; ^2^Department of Hepatobiliary and Pancreatic Surgery, The First Affiliated Hospital of Zhengzhou University, Zhengzhou, China; ^3^Institute of Hepatobiliary and Pancreatic Diseases, Zhengzhou University, Zhengzhou, China; ^4^Zhengzhou Basic and Clinical Key Laboratory of Hepatopancreatobiliary Diseases, The First Affiliated Hospital of Zhengzhou University, Zhengzhou, China; ^5^Department of Cerebrovascular Disease, Zhengzhou University People’s Hospital, Zhengzhou, China

**Keywords:** ferroptosis, hepatocellular carcinoma, tumor microenvironment, molecular subtype, immunotherapy

## Abstract

**Background:**

Ferroptosis is essential for tumorigenesis and progression of hepatocellular carcinoma (HCC). The heterogeneity of ferroptosis and its relationship with tumor microenvironment (TME) have still remain elusive.

**Methods:**

Based on 74 ferroptosis related genes (FRGs) and 3,933 HCC samples from 32 datasets, we comprehensively explored the heterogenous ferroptosis subtypes. The clinical significance, functional status, immune infiltration, immune escape mechanisms, and genomic alterations of different subtypes were further investigated.

**Results:**

We identified and validated two heterogeneous ferroptosis subtypes: C1 was metabolism^low^immunity^high^ subtype and C2 was metabolism^high^immunity^low^ subtype. Compared to C2, C1 owned worse prognosis, and C1 tended to occur in the patients with clinical characteristics such as younger, female, advanced stage, higher grade, vascular invasion. C1 and C2 were more sensitive to immunotherapy and sorafenib, respectively. The immune escape mechanisms of C1 might be accumulating more immunosuppressive cells, inhibitory cytokines, and immune checkpoints, while C2 was mainly associated with inferior immunogenicity, defecting in antigen presentation, and lacking leukocytes. In addition, C1 was characterized by BAP1 mutation, MYC amplification, and SCD1 methylation, while C2 was characterized by the significant alterations in cell cycle and chromatin remodeling processes. We also constructed and validated a robust and promising signature termed ferroptosis related risk score (FRRS) for assessing prognosis and immunotherapy.

**Conclusion:**

We identified and validated two heterogeneous ferroptosis subtypes and a reliable risk signature which used to assess prognosis and immunotherapy. Our results facilitated the understood of ferroptosis as well as clinical management and precise therapy of HCC.

## Introduction

Primary liver cancer is the sixth most prevalent malignant tumor worldwide and ranks fourth among the causes of tumor-related deaths, with approximately 840,000 new cases each year (1). Hepatocellular carcinoma (HCC) is major histological type (75–85%) and characterized by high invasiveness and mortality rate (1). Surgical resection is mainly performed for early HCC, but the 5-year recurrence rate is up to 70%, and most patients relapse within 2 years after surgery (2). Patients with unresectable HCC usually receive the multi-kinase inhibitors such as sorafenib and lenvatinib, but drug-resistance and adverse reactions limit the survival benefit (3). In recent years, although great progress in immunotherapy represented by immune checkpoint inhibitors (ICI), only 25% of patients have durable responses (4, 5). Even when combined with other treatment modalities such as local ablation and transcatheter arterial chemoembolization (TACE), the 5-year survival rate of patients is only 18% (6). Therefore, there is still a long way to improve the therapeutic effect of HCC patients.

Ferroptosis is a newly discovered pattern of programmed cell death characterized by iron-dependent lipid peroxidation and accumulation of reactive oxygen species (ROS), distinguished from typical apoptosis, autophagy, and programmed necrosis (7). Sorafenib, as the first-line drug for advanced HCC, could inhibit cystine-glutamate antiporter (system Xc^−^), and further lead to ferroptosis due to glutathione (GSH) depletion. Our previous studies confirmed that haloperidol could enhance sorafenib-induced ferroptosis in HCC (8), moreover, sigma‐1 receptor can antagonize the ferroptosis in HCC, and non-coding RNAs further regulated the process (9, 10). In addition to iron metabolism, lipid metabolism also plays a pivotal part in ferroptosis. Ou and colleagues found that low density lipoprotein docosahexaenoic acid nanoparticles could induce ferroptosis through glutathione peroxidase-4 (GPX4) inactivation, GSH depletion, and lipid peroxidation, thereby significantly inhibiting the growth of HCC (11). The above suggests that ferroptosis play an essential role in the progression as well as treatment of HCC, and further mining mechanisms will help the development of new therapeutic strategies.

The cancer immunoediting theory suggests that the tumor microenvironment (TME) can identify the body’s dead cells (mainly apoptotic cells) and then clear them by immune system (12). Were ferroptosis cells the same as apoptotic cells? Wen and colleagues found that ferroptosis cancer cells could release high mobility group box 1 (HMGB1) of the damage-associated molecular pattern molecules (DAMPs) family in an autophagy-dependent manner, and then HMGB1 could elicit an inflammatory response upon recognition by pattern recognition receptors (13). Interestingly, previous study demonstrated that tumor cells with autophagy-dependent ferroptosis could release KRAS protein, which was further packaged into exosomes to promote tumor-associated macrophage (TAM) polarization to exert immunosuppressive effects (14). Recent study also found that GPX4 was essential for the survival and expansion of newly activated T cells. The lipid peroxidation of T cells could promote ferroptosis and further contributing to their low immune response rates to infection (15). Nevertheless, most of these scattered studies focused on the link between ferroptosis and individual immune cell, the interaction between TME and ferroptosis have yet to be further deciphered.

With the deepening of ferroptosis studies, its anti-tumor effect has gradually aroused much interest. Wang and colleagues found that CD8+ T cells activated by anti-PD-1 therapy enhanced the lipid peroxidation of tumor cells by releasing interferon gamma (IFN-γ), while the enhanced ferroptosis response could further elevate the immune efficacy (16). In recent years, the advantages of various new materials in cancer prevention and treatment have gradually emerged. Previous studies demonstrated that manganese-doped silica nanoparticle (MnMSN) can deplete GSH, and on-demand drug release can be achieved by loading sorafenib into MnMSN, while dual induction of ferroptosis is achieved by depletion of GSH and inhibition of intracellular GSH synthesis, showing efficient anti-HCC activity (17). Jiang and colleagues observed that a platelet membrane-camouflaged magnetic nanoparticle could sensitize ferroptosis by inhibiting system Xc^−^, which lead to immunosuppressive M2 TAM reversely polarize to the anti-tumor M1 phenotype, further increasing response to immunotherapy (18). Therefore, the more exploration of the ferroptosis heterogeneity might facilitate the target treatment in HCC.

In the present research, we collected a total of 3,933 HCC samples from 32 datasets for analysis. Based on the expression of ferroptosis related genes (FRGs), we identified and validated two heterogeneous subtypes, high and low ferroptosis subtypes, and the two subtypes displayed specific clinical outcomes, immune escape mechanisms, and genomics driver events, respectively. Besides, we developed and validated a prognosis signature termed ferroptosis related risk score (FRRS), FRRS demonstrated outstanding advantages in predicting prognosis and response to immunotherapy. Overall, our work may deepen the understanding of ferroptosis, as well as provide a basis and reference for the clinical management and targeted therapy of HCC.

## Methods and Materials

### Data Source and Processing

The workflow of our study was shown in **Figure S1**. We retrieved eligible datasets from GEO (Gene Expression Omnibus), the Cancer Genome Atlas (TCGA), and the International Cancer Genome Consortium (ICGC) using the following criteria: (1) data was acquired using microarray platforms detecting >10,000 genes; (2) the probe-to-gene mapping annotations were clear; (3) there were >=30 patients in each dataset; (4) patients with primary liver cancer were retained; (5) untreated patients; (6) samples taken after intervention (e.g. after cancer resection) were excluded.

A total of 3,933 eligible HCC samples were enrolled from 32 meta datasets including GSE102079, GSE107170, GSE109211, GSE112790, GSE116174, GSE121248, GSE14323, NCI (National Cancer Institute) cohort (GSE14520), GSE16757, GSE19977, GSE20017, GSE25097, GSE36376, GSE36411, GSE39791, GSE43619, GSE45436, GSE46444, GSE50579, GSE54236, GSE57957, GSE62043, GSE62232, GSE63898, GSE64041, GSE76297, GSE76427, GSE84005, GSE87630, GSE9843, TCGA-LIHC, and ICGC-LIRI-JP. Among them, only NCI, TCGA-LIHC, and ICGC-LIRI-JP datasets possessed completely clinical and prognosis information (**Table S1**). All expression data was log-2 transformed because gene expression data is often heavily right-skewed in the linear scale. We took the gene intersection of all datasets and retained the common 8,731 genes; and all other genes can be considered “missing” for at least one cohort. To our knowledge, there are no guidelines for handling missing data in multicohort studies. However, guidelines for randomized clinical trials recommend skipping imputation and using only observed data when more than 40% of the data is missing. In this study, we served 30 meta cohorts from GEO database as the discovery cohort, and TCGA-LIHC and ICGC-LIRI-JP datasets as two independent validation cohorts.

The rma function implemented in affy package was employed to normalize the raw data from Affymetrix, and normalized matrix files of the other microarrays from other platforms were directly downloaded. Batch correction was performed using the combat algorithm implemented in SVA package. The RNA-seq data (FPKM normalized) of TCGA-LIHC cohort was obtained from the UCSC-Xena database and was further transformed to log2 (TPM+1). The RNA-seq data of ICGC-LIRI-JP dataset was retrieved from the ICGC data portal. Subsequently, we transformed the expression data into z-score in both discovery and validation cohorts. The corresponding clinical information were obtained from GEO, UCSC, and ICGC databases. The somatic mutation, copy number variation (CNV), and DNA methylation data in TCGA-LIHC were all downloaded from the TCGA portal. We calculated or recruited the tumor mutation burden (TMB), single nucleotide variants (SNV) and indel neoantigen load, microsatellite instability (MSI), cancer testis antigen (CTA) scores, and TCR/BCR diversity from Thorsson et al. study (19).

### Identification of the Ferroptosis Subtypes of HCC

After a detailed literature research, we selected a total of 74 FRGs (**Table S2**). According to the FRGs expression, we performed consensus clustering in the discovery cohort *via* ConsensusClusterPlus package (20). The method was set to Kmeans algorithm based on the Euclidean distance, 1,000 times iteration, and taking 80% of the samples for each iteration. The number of clusters was set from 2 to 9, and the optimal number was determined by the cumulative distribution function (CDF) of the consensus score and the proportion of ambiguous clustering (PAC) (21). The NbClust package was applied to further verify the optimal number (22). Principal component analysis (PCA) was used to distinguish different subtype information in two-dimensional space.

### Validation of the Ferroptosis Subtypes

We further quantitatively assessed the stability and reproducibility of proposed subtypes in the discovery and validation cohorts with in group proportion (IGP) statistic (23). IGP was defined as the proportion of the nearest neighbors of a certain subtype sample that were also assigned to the same subtype. A high IGP indicated that samples of this subtype were reproducible partitioned. To measure the IGP, we first calculated the centroid of each subtype in the discovery cohort. Each sample in the TCGA and ICGC validation cohorts was assigned to a certain subtype with the highest Pearson correlation coefficient between centroid and sample. The permutation in the clusterRepro package was set to 2000.

### Functional Analysis and Immune Cell Infiltration Assessment

The gene set variation analysis (GSVA) was performed to identify specific pathways of each subtype (24). We downloaded Hallmark and KEGG gene sets from the Molecular Signatures Database and further transformed the gene expression matrix into gene set matrix using the GSVA package. Afterwards, we performed gene sets difference analysis using the limma package and the screening threshold were set to |log2 fold change (FC)| >0.2 and adjusted P-value <0.05. Adjusted P-value was obtained from the Benjamini–Hochberg multiple test correction.

Referring to Charoentong et al. study (25), we obtained the markers of 23 immune cells including: innate immune cells (activated dendritic cells, CD56+ natural killer cells, CD56− natural killer cells, eosinophils, immature dendritic cells, macrophages, mast cells, MDSC, monocytes, natural killer cells, neutrophils, and plasmacytoid dendritic cells) and adaptive immune cells (activated B cells, activated CD4+ T cells, activated CD8+ T cells, Gamma delta T cells, immature B cells, natural killer T cells, Treg cells, follicular helper T cells, Th1 cells, Th2 cells, and Th17 cells). Endothelial cells and fibroblasts, also the important components of TME, played a crucial role in tumor inflammation, angiogenesis, invasion, and metastasis. The markers of endothelial cell and fibroblast were retrieved from the MCP-counter (26) (**Table S3**). Based on these markers, we applied the single sample gene set enrichment analysis (ssGSEA) algorithm to evaluate the infiltration abundance of 25 TME cells.

### Assessing Clinical Significance of the Ferroptosis Subtypes

We compared the differences between the two subtypes in age, gender, Body Mass Index (BMI), AJCC stage, grade and vascular invasion, and estimated relapse-free survival (RFS) and overall survival (OS) by the Kaplan-Meier survival analysis. Afterwards, we applied the pRRophetic package to predict the sensitivity to sorafenib in both discovery and validation cohorts (27). The IC50 (half maximal inhibitory concentration) values of the two subtypes were estimated by ridge regression, the smaller its IC50, the more sensitive it was to the drug. In addition, we also utilized TIDE web tool (http://tide.dfci.harvard.edu) to predict the sensitivity of the two subtypes to immunotherapy (28). TIDE algorithm was a computational method to model two primary mechanisms of tumor immune evasion: the induction of T cell dysfunction in tumors with high infiltration of cytotoxic T lymphocytes (CTL) and the prevention of T cell infiltration in tumors with low CTL level. The Subclass mapping algorithm was used to evaluate the similarity of gene expression patterns between the two subtypes and immunotherapy-sensitive/insensitive populations (29).

### Deciphering the Genomic Variation Landscape of the Two Subtypes

We identified significantly mutated genes (SMGs) in the two subtypes using MutSigCV 1.41 software, and genes with q values <0.05 were retained to further analysis. The MutationalPatterns package was applied to extract the mutational signatures of each subtype, and non-negative matrix factorization (NMF) determined the optimal number of mutational signatures. It turned out that the optimal number was 3 in both subtypes (**Figures S8K, L**). We then calculated the cosine similarity metrics between these extracted mutational signatures and 30 mutational signatures from the COSMIC database, and named after the most similar COSMIC signature. The GISTIC 2.0 software in GenePattern was applied to identify significantly amplified or deleted broad and focal segments. The global methylation level (GML) was retrieved from Jung et al. study (30). Moreover, we performed the following procedure to identify epigenetically silenced genes (ESGs): (1) excluding the CpG sites methylated in normal tissues (mean β-value of >0.2); (2) the DNA methylation data was divided into the methylation group and unmethylation group, according to the cutoff (β-value = 0.3), and further removed the probe that less than 10% of the tumor samples in the methylated group; (3) for each probe, if the difference between the corresponding gene mean expression in the unmethylated group and that in the methylated group was >1.64 standard deviations of the unmethylated group, the probe would be labeled as epigenetically silenced; (4) when multiple probes were assigned to the same gene, the gene with more than half of the corresponding probes were labeled as epigenetically silenced, and identified as ESG.

### Generation of Ferroptosis Related Risk Score

We applied the limma package to identify differentially expressed genes (DEGs) between the two subtypes, setting the thresholds: |log2 FC| > 1 and adjusted P-value < 0.05. Adjusted P-value was obtained from the Benjamini–Hochberg multiple test correction. Combined with the previously obtained significant CNV associated genes (CAGs), SMGs and ESGs, we used Venn diagram to illustrate the relationship among the four gene sets, and then selected genes present in at least two gene sets for further analysis. A univariate Cox regression analysis revealed the prognosis value of these genes. The genes with statistically significant (p < 0.05) were incorporated into multivariate Cox regression analysis. Afterwards, we constructed the ferroptosis related signature using stepwise regression, and selected the optimal model when the AIC (Akaike Information Criterion) score was the smallest. This optimal model was as follows:

risk score=∑(Expression(gene)∗coef(gene))

where expression (gene) denoted the expression level of a gene and coef (gene) represented its regression coefficient. We named the signature the ferroptosis-related risk score (FRRS). The HCC samples were categorized into high and low FRRS groups according to the optimal cut-off value determined by the survminer package. Then, we performed Kaplan-Meier analysis of FRRS in three independent cohorts: TCGA, ICGC, and NCI, and further assessed the predictive accuracy of model with Concordance index (C-index).

### Collection of Immunotherapy Cohorts and Biomarkers

We systematically collected immunotherapeutic cohorts that were publicly available and had expression data and complete clinical information, and three cohorts finally enrolled in our study: (1) advanced urothelial cancer patients who received the intervention of anti-PD-L1 antibody atezolizumab (IMvigor210 cohort) (31); (2) metastatic melanoma treated with anti-PD-1 antibody pembrolizumab (GSE78220 cohort) (32); (3) melanomas received adoptive T cell therapy (GSE100797 cohort) (33). According to the RECIST v1.1 criterion, patients whose treatment effectiveness could not be assessed were excluded. The complete response and partial response were regarded as immunotherapy response, the stable disease and progressive disease were regarded as immunotherapy non-response. The normalized expression data was further transformed into z-score. We evaluated the predictive performance of FRRS in three immunotherapy cohorts, and compared FRRS with seven other known biomarkers, including TMB, TIDE, MSI score, Merck18, IFGN, CD8, and CD274 (28, 34–36) (**Table S4**). The receiver operator characteristic (ROC) curves and the area under the ROC curve (AUC) were applied to estimate the predictive accuracy of each biomarker.

### Statistical Analysis

The Pearson’s chi-squared test or Fisher’s exact test was employed to compare categorical variables. Continuous variables were compared between two groups through Wilcoxon rank-sum test or T test. Survival analysis including Kaplan-Meier and Cox regression analysis was performed by survival R package. The optimal cut-off value was determined by survminer R package. The ROC for predicting immunotherapy was performed by pROC R package. All P value were two-side, with p < 0.05 as statistically significance. The whole data processing, statistical analysis, and plotting were conducted in R 3.6.3 software.

## Results

### Genomic Variation Landscape of FRGs in HCC

**Figure 1:**
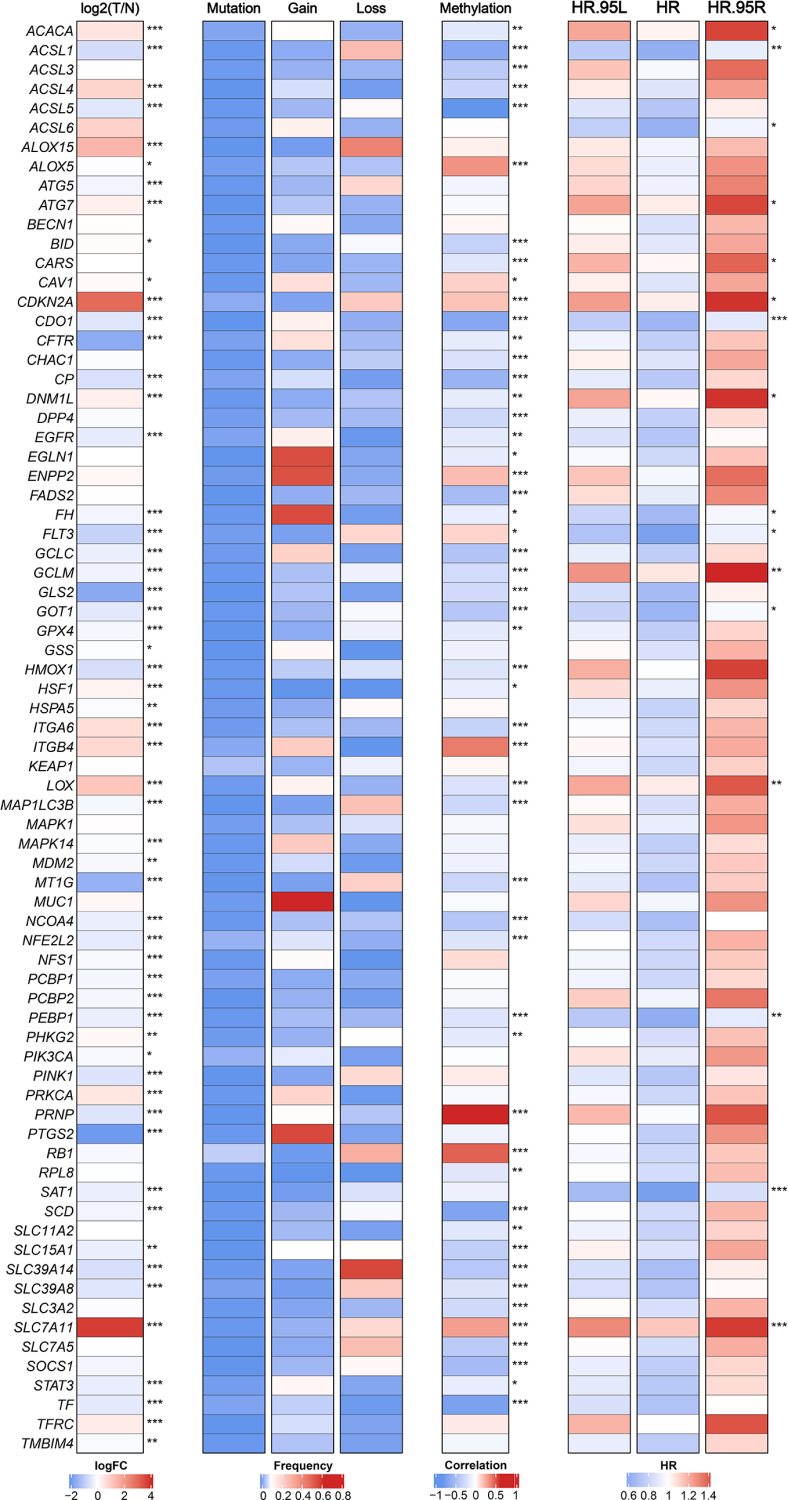
The expression, genomic variation and hazard ratios of FRGs in TCGA-LIHC. From left to right panel, the expression difference of FRGs in tumor tissues compared with normal tissues, the mutation and copy number variation frequency of FRGs, the correlation of DNA methylation modifications and expression for FRGs, and univariate Cox regression analysis presented hazard ratios of FRGs. *p < 0.05, **p < 0.01, ***p < 0.001.

We retrieved 74 FRGs from previous literatures and KEGG pathways (**Table S2**). The multi-omics landscape of FRGs were summarized from the TCGA-LIHC cohort (**Figure 1**). According to these genes, we can separate tumor tissue from normal tissue distinctly (**Figure S2A**). Most of FRGs displayed significant expression differences between tumor and normal tissues. For instance, SLC7A11, CDKN2A, and ALOX15 were up-regulated in HCC, while PTGS2, CFTR, and GLS2 were down-regulated. Further studies observed infrequent mutations of FRGs and widespread copy number variations (CNVs), which suggested that CNVs might play a dominant role in the regulation of FRGs relative to mutation. For example, EGLN1, ENPP2, and MUC1 focused on amplification of copy number, whereas SLC39A14, ALOX15, and ACSL1 preferred deletion. Besides, the DNA methylation also displayed a broad regulatory effect on FRGs, such as ACSL1, ACSL5, and SCD. Univariate Cox regression analysis further demonstrated that most of FRGs played a protective role in HCC, which in line with the protective biological function of FRGs (**Figure 1**).

### Identification and Validation of the Ferroptosis Subtypes

**Figure 2:**
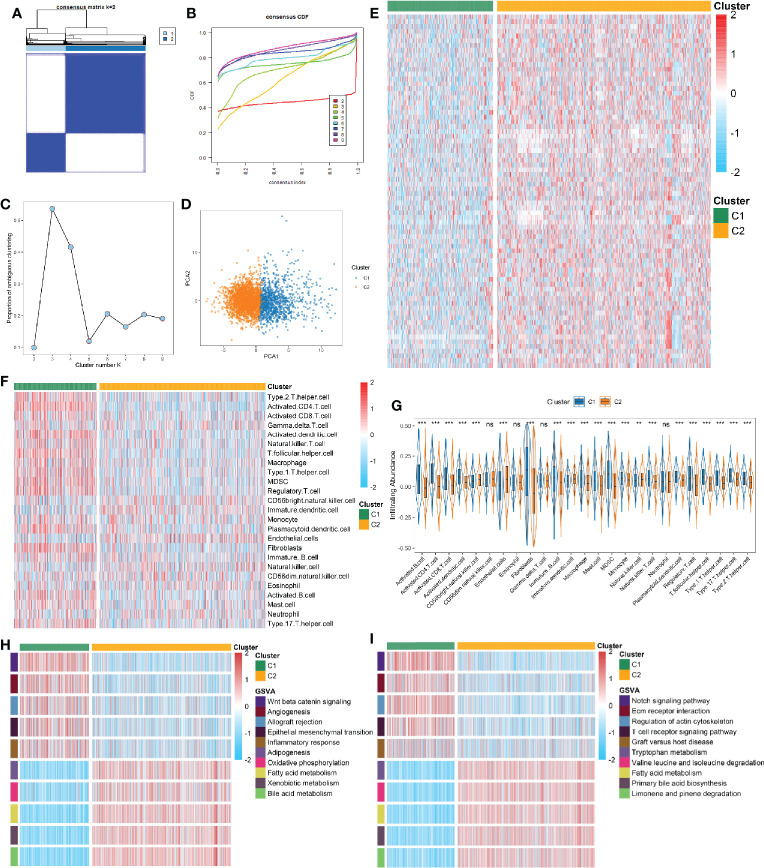
**(A)** The consensus score matrix of all samples when k = 2. A higher consensus score between two samples indicates they are more likely to be grouped into the same cluster in different iterations. **(B)** The cumulative distribution functions of consensus matrix for each k (indicated by colors). **(C)** The proportion of ambiguous clustering (PAC) score, a low value of PAC implies a flat middle segment, allowing conjecture of the optimal k (k = 2) by the lowest PAC. **(D)** Two-dimensional principle component plot by the expression of 74 FRGs in the two subtypes. The orange dots represented C1, and blue dots represented C2. **(E)** The expression heatmap of 74 FRGs in the two subtypes. **(F)** The heatmap of immune cells in the two subtypes. **(G)** The infiltration difference of TME cells between the two subtypes. The asterisks represented the statistical p value (^ns^P > 0.05; **P < 0.01; ***P < 0.001). **(H, I)** GSVA enrichment analysis revealed activated Hallmark **(H)** and KEGG **(I)** pathways of the two subtypes.

A total of 3,327 samples from 30 GEO datasets were defined as the discovery cohort, and further divided into k groups (k = 2 ~ 9) *via* ConsensusClusterPlus R package. We found that k = 2 was optimal choice according to the CDF curve of the consensus score (**Figures 2A, B**). The PAC and NbClust methods further verified the result (**Figures 2C** and **S2B**). The principal component analysis of 74 FRGs expression showed significant separation between two clusters (**Figure 2D**). To ensure the reliability and stability of the clustering results from the meta cohorts, we further performed IGP analysis in two independent cohorts. The results exhibited that the IGP values of C1 was 90.3% and C2 was 92.9% in the TCGA cohort, while was 88.4% and 91.7% in the ICGC cohort (all p < 0.001). The NbClust also indicated it was optimal to split into two clusters in both cohorts (**Figures S2C, D**).

Compared to C1, most of FRGs were significantly up-regulated in the C2 (**Figure 2E**). Recent studies revealed that ferroptosis can induce tumor-specific immune responses and enhance the effect of immunotherapy (18, 37). Further correlation analysis suggested intense correlations between 74 FRGs and TME cells in HCC (**Figure S2E**). We then explored the differences of TME cells infiltration in the two subtypes. It turned out C1 exhibited a higher overall level of infiltration (**Figure 2F**). In addition to display superior immune activated cells (e.g., CD 4+/CD8+ T cells), C1 also showed higher abundance of immunosuppressive cells (e.g., Treg, MDSC, Th17 cell, and fibroblast) (**Figure 2G**). The above implied that ferroptosis may have a profound impact on TME in HCC. To further clarify the biological characteristics of the two subtypes, we performed GSVA enrichment analysis using Hallmark and KEGG gene sets. As illustrated, C1 was observably enriched in inflammation related pathways, such as allograft rejection, inflammatory response, and T cell receptor signaling pathway; while C2 was predominantly associated with metabolism related pathways, such as oxidative phosphorylation, fatty acid metabolism, bile acid metabolism, and amino acid metabolism (**Figures 2H, I**). The similar results were obtained from the TCGA and ICGC cohorts (**Figures S3, S4**). Overall, the two subtypes were defined as follows: 1) metabolism^low^immunity^high^type (LMHI): low levels of FRGs expression and inflammation-related pathways enrichment as well as high abundant of immune cells infiltration; 2) metabolism^high^immunity^low^ type (HMLI): high levels of FRGs expression and metabolism-related pathways enrichment as well as low abundant of immune cells infiltration.

### Clinical Characteristics of the Ferroptosis Subtypes

**Figure 3:**
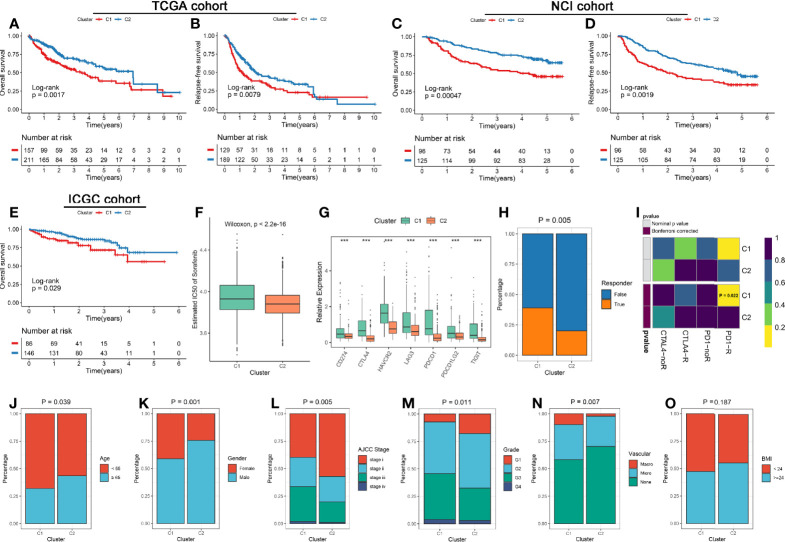
**(A, B)** Kaplan-Meier analysis for OS **(A)** and RFS **(B)** of the two subtypes in the TCGA cohort. **(C, D)** Kaplan-Meier analysis for OS **(C)** and RFS **(D)** of the two subtypes in the NCI cohort. **(E)** Kaplan-Meier analysis for OS in the ICGC cohorts. **(F)** The estimated IC50 of sorafenib between the two subtypes in the discovery cohorts. **(G)** Comparison of ICP molecules expression between the two subtypes. The asterisks represented the statistical p value (***P < 0.001). **(H)** The TIDE algorithm was used to predict the sensitivity of the two subtypes to immunotherapy in the discovery cohorts. **(I)** Submap analysis of the two subtypes and 47 pretreated patients with comprehensive immunotherapy annotations in the discovery cohorts. For Submap analysis, a smaller p-value implied a more similarity of paired expression profiles. **(J–O)** Composition percentage of the two subtypes in clinical characteristics such as age **(J)**, gender **(K)**, BMI **(L)**, AJCC stage **(M)**, grade **(N)**, and vascular invasion **(O)**.

The clinical significance of two subtypes were further explored. Survival analysis revealed C2 had a better OS and RFS relative to C1 in three cohorts (**Figures 3A–E**). Previous studies indicated sorafenib could induce ferroptosis by inhibiting System Xc^-^ (38). We thus predicted the sensitivity of two subtypes to sorafenib using the pRRophetic package, and the result prompted that C2 was more likely to benefit from sorafenib (**Figure 3F** and **Figures S5A, D**). Besides, the previous analysis displayed C1 possessed superior immune cells infiltration, the checkpoint molecules (e.g., PD-L1 and CTLA-4) also were over-expressed in C1 (**Figure 3G**). These results hinted C1 may be more sensitive to immunotherapy. Therefore, we further assessed the effectiveness of immunotherapy on both subtypes. Using the TIDE web tool, C1 displayed a higher response compared to C2, and similar results was obtained in the two validation cohorts (**Figure 3H** and **Figures S5B, E**). Moreover, the Submap algorithm were applied to evaluate the similarity of expression profiles between the two subtypes and 47 pretreated patients with comprehensive immunotherapy information, and the results indicated C1 was significantly related to patients responding to anti-PD-1 treatment, and similar results was obtained in the two validation cohorts (**Figure 3I** and **Figures S5C, F**). In addition, we also observed that patients in C1 subtype was significantly associated with the features such as younger, female, advanced stage, higher grade, and vascular invasion (**Figures 3J–N**). There was no difference of BMI between two subtypes (**Figure 3O**).

### Potential Extrinsic Immune Escape Mechanism of the Two Subtypes

We questioned whether the effect of ferroptosis on HCC could cause the differences in immune escape mechanisms between the two subtypes. Therefore, we first researched the extrinsic immune escape mechanism (12). Previous publications have shown that extrinsic immune escape mainly includes four aspects: lack of leukocytes, massive immunosuppressive cells, high concentrations of immunosuppressive cytokines, and increase in fibroblasts (39).

**Figure 4:**
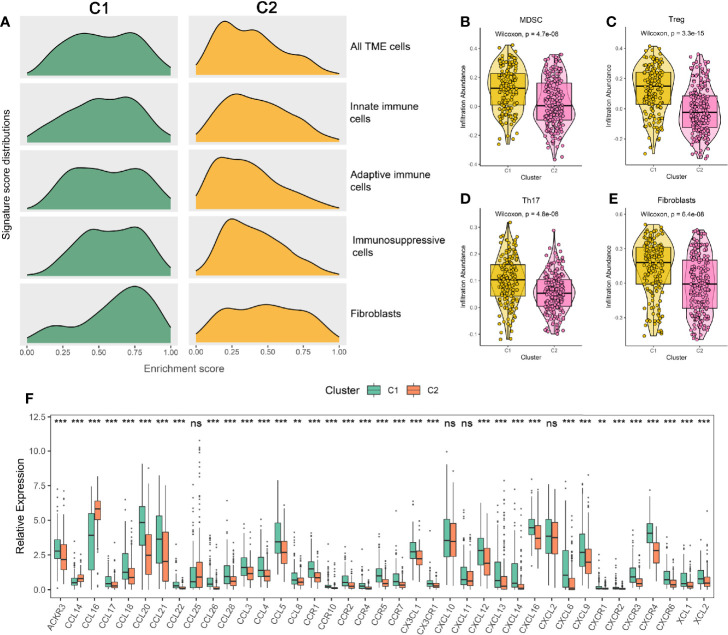
**(A)** Signature score distributions of five cell subsets between the two subtypes. **(B–E)** Comparison of MDSC **(B)**, Treg **(C)**, Th17 **(D)**, and fibroblasts **(E)** between the two subtypes. **(F)** The relative expression levels of chemokines and their ligands of the two subtypes. The asterisks represented the statistical p value (^ns^P > 0.05; **P < 0.01; ***P < 0.001).

According to the above results, we summarized the abundance distribution of TME cells in the two subtypes. As shown in **Figure 4A**, the abundance of immunosuppressive cells and fibroblasts in C1 were superior, while C2 demonstrated a lack of innate immune cells and adaptive immune cells. In addition, the infiltration levels of immunosuppressive cells such as MDSC, Treg, Th17, and fibroblasts were also higher in C1 (**Figures 4B–E**). Consistent with these results, C1 also exhibited an increase in chemokines, interleukins, interferons, and other important cytokines and their receptors, such as CCL5 (recruiting MDSC to migrate to tumor areas), IL-10 (a cytokine synthesis inhibitor), and TGF-β3 (having a wide range of immunosuppressive activities) (40–42) (**Figure 4F** and **Figures S6A, B**). Overall, we speculated that the aggregation of immunosuppressive cells, fibroblasts, and the high concentrations of immunosuppressive cytokines might lead to the extrinsic immune escape of C1, while C2 was mainly related to immune cells defects.

### Potential Intrinsic Immune Escape Mechanism of the Two Subtypes

**Figure 5:**
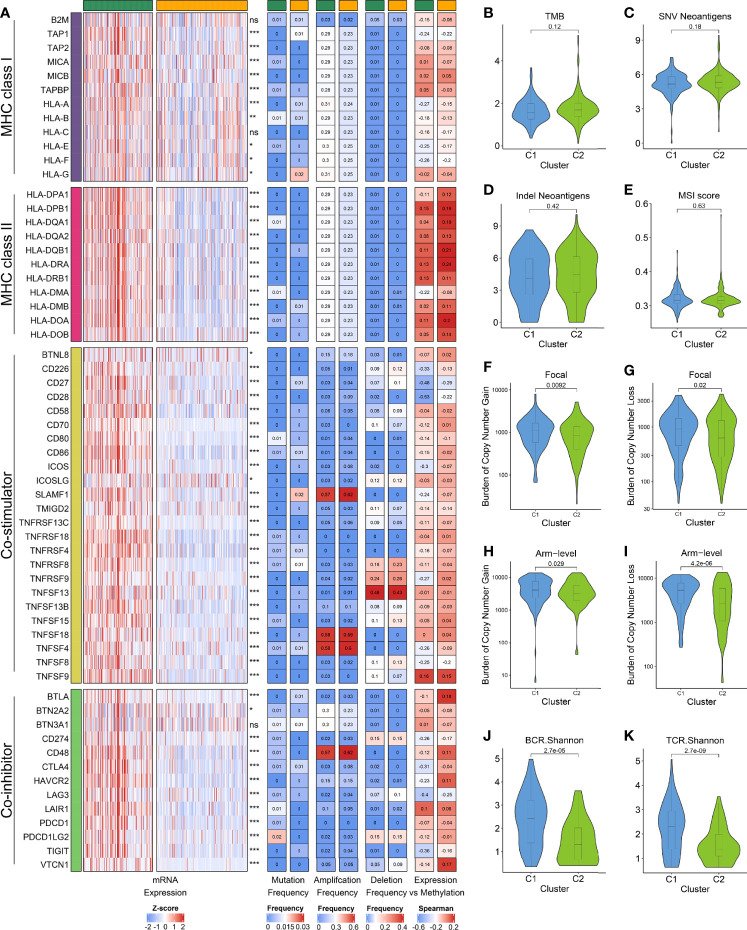
**(A)** From left to right: mRNA expression; mutation frequency; amplification frequency; deletion frequency, and expression *versus* methylation (gene expression correlation with DNA methylation β value) for MHC molecules, co-stimulators and co-inhibitors in the two subtypes. **(B–E)** Comparison of the two subtypes in four immunogenicity associated indicators such as TMB **(B)**, SNV neoantigens **(C)**, indel neoantigens **(D)**, and MSI score **(E)**. **(F–I)** Comparison of the two subtypes in focal **(F, G)** and broad **(H, I)** CNV burden. **(J, K)** The distribution of TCR **(J)** and BCR **(K)** diversity in the two subtypes.

We next investigated the potential intrinsic immune escape mechanism in HCC, including the following three aspects: antigen presentation capacity, expression of immune checkpoints (ICPs), and tumor immunogenicity (12). Compared to C1, the expression of MHC and APS were significantly lower in C2, suggesting that defective antigen presentation capacity might be an intrinsic immune escape mechanism for C2 (**Figure 5A** and **Figures S7A, B**). Subsequently, we explored the expression and regulatory patterns of the immune checkpoints in the two subtypes. C1 displayed the higher expression of costimulatory and coinhibitory molecules, which implied that C1 might overexpress immune checkpoints (e.g., CTLA4, CD274, PDCD1) to evade the immune elimination after immune activation (**Figure 5A** and **Figures S7C, D**). Notably, the expression difference of ICPs were not derived from mutation, but were strongly associated with CNV and methylation. For example, TNFSF4, TNSF18 and CD48 focus on amplification, whereas TNFSF13 possessed a high frequency of deletion (**Figure 5A**). The DNA methylation of CD28, CD27, and LAG3 obviously negatively regulated their expression, implying epigenetic silencing (**Figure 5A**). Therefore, CNV and methylation modification might play a dominant role in regulating ICPs compared to mutation, which pointed a new direction for the development of immune checkpoint inhibitors (ICIs).

Afterwards, we focused on evaluating eight indicators related to HCC immunogenicity. As the main source of tumor-specific antigens (43), TMB, neoantigen load (including SNV neoantigens and indel neoantigens), and MSI status had no significant difference between the two subtypes, while C1 displayed the higher CTA score (**Figures 5B–E** and **Figure S7E**). Besides, we found that C1 has evidently higher CNV load in the level of focal, chromosomal arm and base, respectively (**Figures 5F–I** and **Figure S7F**). In line with this, the TCR/BCR diversity were superior in C1 (**Figures 5J, K** and **Figures S7G, H**). These results suggested C1 possessed higher immunogenicity relative to C2, and CNV may dominate the differences in immunogenicity of the two subtypes.

### Comprehensive and Integrative Genomic Characterization of the Two Subtypes

**Figure 6:**
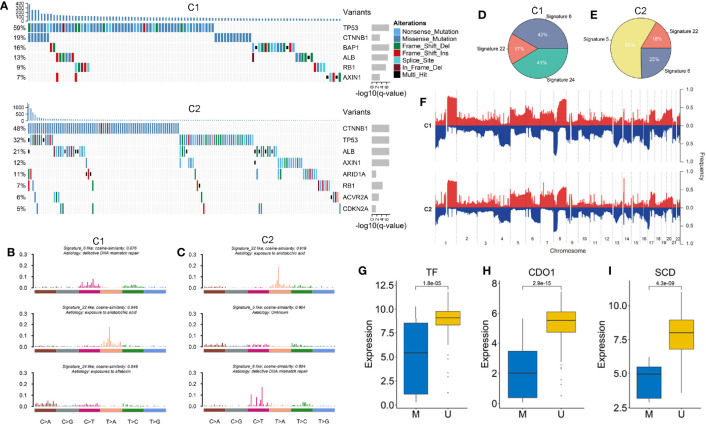
**(A)** The waterfall plot of significantly mutation genes in the two subtypes. Each column represented individual patients. The upper barplot showed TMB, the number on the left showed the proportion of samples with mutations. The right barplot indicated the mutation frequency in each gene. **(B, C)** The three mutation signatures with the highest cosine similarity to COSMIC signatures in C1 **(B)** and C2 **(C)**. The etiology of each signature and the cosine similarity between the original and the reconstructed mutation signatures were indicated. **(D, E)** The pie charts showed the proportion of the three mutation signatures contributing to the mutations spectrum of C1 **(D)** and C2 **(E)**. **(F)** The copy number variations of the two subtypes. **(G–I)** The expression difference of three ESGs including TF, CDO1 and SCD between the methylated and unmethylated groups.

Based on the MutSigCV algorithm, a total of nine SMGs was identified in the two subtypes (**Figure 6A** and **Figures S8A, I**). We observed the mutation of these genes had an influenced on their expression such as CTNNB1, AXIN1, and RB1. Univariate Cox regression further revealed the prognostic value of SMGs (**Figure S8J**). The two subtypes shared five common SMGs including TP53, CTNNB1, ALB, RB1 and AXIN1, suggesting their mutations were prevalent in HCC. Specifically, tumor suppressor BAP1 was a SMG of C1, while SMGs related to chromatin remodeling such as ARID1A, ACVR2A, and CDKN2A mainly occurred in C2 (44, 45). In addition, we further explored the mutation signatures of the two subtypes and found that signature 6 (associated with defective DNA mismatch repair) and signature 22 (had a history of exposure to aristolochic acid) presented in both subtypes, but with different proportions (**Figures 6B–E**). Notably, we also discovered that signature 24 associated with aflatoxin was specifically presented in C1, whereas age-related signature 5 only existed in C2 (**Figures 6B, C**). Overall, C1 was mainly dominant in signature 6 and signature 22, while signature 5 had a master proportion in C2, indicating that different leading carcinogenic factors in the two subtypes (**Figures 6D, E**).

GISTIC2.0 was utilized to define recurrently amplified and deleted regions in the two subtypes (**Figure 6F** and **Table S5**). The results showed that the two subtypes had frequent CNVs in the regions where oncogenes and tumor suppressor genes (e.g. MYC and TP63), as well as cell cycle regulators (e.g. CDK3, CDK8, and MAPK11) were located, which indicated the CNVs might have a profound impact on the tumorigenesis and progression of HCC. We observed recurrent focal CNVs in C1 included amplifications containing 8q24.21 (MYC) and 1p11.2 (NOTCH2) and deletion of 10q23.1(GRID1). Recurring focal CNVs in C2 included amplifications of 6p21.1 (VEGFA) and 17q25.1 (CDK3), and deletion of 3q28 (TP63), 13q13.3 (BRCA2, CDK8), and 22q13.33 (MAPK11). These specific CNVs might contribute to formation of the two subtypes.

We further investigated methylation modification in the two subtypes and found that C1 had a higher GML than C2 (**Figure S9A**). Next, we identified 30 and 17 ESGs from C1 and C2, respectively (**Figures S9B, C**). Among them, we observed that the expression levels of three FRGs (e.g. TF, CDO1, and SCD) were significantly lower in methylated group (**Figures 6G–I**). Notably, both subtypes possessed a common ESG, HOXA3, which was associated with focal adhesion and ECM-receptor (46). We also discovered some specific ESGs such as ACOX2 and SCD that played a crucial role in lipid metabolism only appeared in C1. This might explain that C1 was a hypometabolic status. Whereas WIPF3 and LAMA3 that associated with pathogen infection and inflammatory diseases specifically presented in C2. These ESGs might lead to defects in TME cells and cytokines in C2.

### A Novel Prognostic and Immunotherapy Biomarker: FRRS

**Figure 7:**
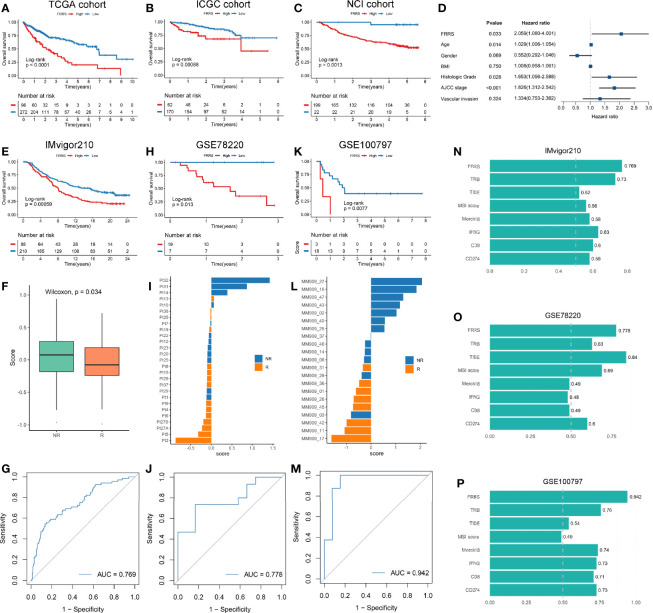
**(A–C)** Kaplan-Meier survival analysis of high FRRS and low FRRS group in TCGA **(A)**, ICGC **(B)**, and NCI **(C)** cohorts. **(D)** FRRS and clinical factors were combined for multivariate Cox regression analysis. **(E–G)** Kaplan-Meier survival analysis of high FRRS and low FRRS groups **(E)**, the distribution of FRRS between response and nonresponse groups **(F)**, and ROC curve of the FRRS signature for predicting immunotherapy response **(G)** in IMvigor210 cohort. **(H–J)** Kaplan-Meier survival analysis of high FRRS and low FRRS groups **(H)**, the distribution of FRRS between response and nonresponse groups **(I)**, and ROC curve of the FRRS signature for predicting immunotherapy response **(J)** in GSE78220 cohort. **(K–M)** Kaplan-Meier survival analysis of high FRRS and low FRRS groups **(K)**, the distribution of FRRS between response and nonresponse groups **(L)**, and ROC curve of the FRRS signature for predicting immunotherapy response **(M)** in GSE100797 cohort. **(N–P)** AUC values of FRRS and seven other biomarkers for predicting the immunotherapy response in IMvigor210 **(N)**, GSE78220 **(O)**, and GSE100797 **(P)** cohorts.

We identified 1,023 DEGs between the two subtypes (**Figure S10A**). GO enrichment analysis showed that these genes were strongly correlated with extracellular matrix organization and organic acid transport, and KEGG pathway analysis revealed that cytokine-cytokine receptor interaction, bile secretion, and Wnt signaling pathway were significantly enriched (**Figure S10B**). Among the four gene sets including DEGs, SMGs, CAGs, and ESGs (**Figure S10C**), we selected 33 genes that were present in at least two of the four categories for further study (**Table S6**). Univariate COX regression analysis indicated that six genes had predominant prognostic significance (p < 0.05). Next, we enrolled the six genes (p < 0.05) for multivariate COX regression analysis, a stepwise regression approach was applied. Based on the smallest AIC value, we determined the best model: FRRS = 0.348 * Expression (SLC16A3) − 0.151 * Expression (CPS1). Survival analysis exhibited patients with high FRRS had a worse prognosis (HR: 2.511 [2.145–2.876] in the TCGA cohort, 1.542 [1.236–1.847] in the ICGC cohort, and 1.614 [1.351–1.877] in the NCI cohort) (**Figures 7A–C** and **Figure S11A**). The concordance index (C-index) analysis also confirmed that FRRS had high accuracy in the three independent cohorts of TCGA, ICGC, and NCI (C-index = 0.785; 0.716; 0.733; respectively; **Figure S11B**). Combined with clinical factors, we observed FRRS was an independent prognosis factor in HCC through multivariate Cox regression analysis (**Figure 7D**).

Although immunotherapy represented by ICIs has been gradually recognized as a promising tumor treatment, only a small number of patients can benefit from it (47). We explored the biological characteristics of FRRS related to immunotherapy response, and found that FRRS was significantly positively correlated with the expression of ICP molecules such as HAVCR2, CTLA4, and PDCD1, as well as the infiltration patterns of Treg cells and MDSC (**Figures S11C, D**). Thus, we included three immunotherapy cohorts to further investigated whether FRRS could predict responsiveness of the patients to immunotherapy. In line with the above, patients with high FRRS showed an unfavorable survival in these three cohorts (**Figures 7E, H, K**). In addition, patients who were clinically responsive to immunotherapy showed lower FRRS, suggesting that patients with lower FRRS were more likely to benefit from immunotherapy (**Figures 7F, I, L**). The area under the curve (AUC) for the ROC curve was used to measure the accuracy of FRRS in predicting the response to immunotherapy. These results strongly suggested that FRRS was a reliable biomarker (IMvigor210: AUC = 0.769; GSE78220: AUC = 0.778; GSE100197: AUC = 0.942; **Figures 7G–M**). Then we calculated seven widely used immunotherapy biomarkers, including TMB, TIDE, MSI score, Merck18, IFGN, CD8, and CD274. In all three cohorts, FRRS afforded greater accuracy in the prediction of immunotherapy (**Figures 7N–P**). Notably, TIDE performed worse in predicting response to immunotherapy in the IMvigor210 cohort and GSE100797 cohort (AUC = 0.52 and 0.54; respectively), although the predictive power of FRRS in the GSE78220 cohort is slightly lower than that of TIDE (**Figures 7N–P**). Overall, our study strongly confirmed that FRRS can be used to assess the prognosis and immunotherapy response of patients, and outperformed widely used biomarkers.

## Discussion

Ferroptosis, as a recently recognized programmed cell death modality, has been confirmed to be significantly associated with tumor progression, immune status, and anti-tumor response, and its role in HCC has gradually attracted people’s attention (48, 49). Our study identified and validated two heterogeneous ferroptosis subtypes in HCC. C1 possessed low levels of FRGs expression and high abundance of innate and adaptive immune cells, and were closely associated with inflammation, which was defined as the metabolism^low^immunity^high^ subtype. C2 expressed high FRGs expression but lacked infiltrating immune cells, presented a metabolism-related functional characteristic, which was defined as the metabolism^high^immunity^low^ subtype. We also validated the stability and reproducibility of the two subtypes in two independent cohorts. The two subtypes also exhibited heterogeneity in immune escape mechanisms, genome-driven events, and clinical outcomes (**Table 1**). In addition, based on the two subtypes, we proposed a prognosis signature: FRRS, which was an independent prognosis factor for HCC. Further immunotherapy prediction also indicated FRRS might be a promising immunotherapy marker. These results facilitated the understood of ferroptosis as well as clinical management and precise therapy of HCC.

**Table 1:** Summary of FRGs expression, TME cells infiltration, biological and clinical characteristics, immune escape mechanisms, and genome-driven events for the two ferroptosis subtypes.

Subtype	Cluster 1	Cluster 2
**FRGs expression**	lower	higher
**TME cells infiltration**	higher	lower
**Biological characteristics**	inflammation	metabolism
**Dominant clinical characteristics**		
Prognosis	worse	better
Age	younger	older
Gender	female	male
Stage	more advanced	less advanced
Grade	senior	junior
Vascular invasion	macro or microvascular	none
Sensitivity to sorafenib	lower	higher
Sensitivity to immunotherapy	higher	lower
**Extrinsic immune escape mechanism**		
All TME cells	higher	lower
Innate immune cells	higher	lower
Adaptive immune cells	higher	lower
Immunosuppressive cells	higher	lower
Fibroblasts	higher	lower
**Intrinsic immune escape mechanism**		
MHC expression	higher	lower
APS score	higher	lower
ICPs expression	higher	lower
Immunogenicity	higher	lower
TMB	had no significant difference	
Neoantigen load		
MSI status		
CTA score	higher	lower
CNV load	higher	lower
TCR/BCR diversity	higher	lower
**Cluster-specific genomic variation landscape**		
Mutations	BAP1	ARID1A, ACVR2A,and CDKN2A
Copy number amplifications	8q24.21 (MYC);1p11.2 (NOTCH2)	6p21.1(VEGFA);17q25.1 (CDK3)
Copy number deletions	10q23.1(GRID1)	3q28 (TP63);13q13.3 (BRCA2,CDK8); 22q13.33 (MAPK11)
DNA methylation		
GML	higher	lower
ESGs	ACOX2; SCD	WIPF3; LAMA3

The two subtypes demonstrated distinct clinical characteristics. We observed C1 owned worse OS and RFS relative to C2. In addition, C1 was more prone to occur in the patients with clinical characteristics such as younger, female, advanced stage, higher grade, vascular invasion relative to C2. Further predictions for sorafenib displayed the drug sensitivity of C2 was higher than C1, which might be due to the overexpression of FRGs that could be targeted by sorafenib in C2 (38). Conversely, C1 displayed superior response to immunotherapy. These results might facilitate personalized treatment for patients with HCC.

We then explored the specific immune escape mechanisms of the two subtypes. The TME of C1 accumulated more immunosuppressive cells and inhibitory cytokines, and its overexpressed ICPs could evade immune recognition and clearance after activation. C2 had a lower abundance of immune killer cells, which might arise from its inferior immunogenicity and antigen presentation capacity. These results provided critical references for immunotherapy of HCC. In addition, we also observed that CNV and DNA methylation might play a master role in regulating immunoregulatory factors compared to mutations, which points out the directions for the development of ICIs.

Next, in order to depict the molecular characteristics of the two subtypes, we separately investigated the distinct genome alterations of the two subtypes. As a particular SMG of C1, BAP1 has been certified to block cystine uptake by inhibiting the expression of SLC7A11, leading to lipid peroxidation and ferroptosis, thereby inhibiting tumor progression (50). However, the mutation of BAP1 deprived the above ability, which might partially explain its poor prognosis to some extent. Consistent with the immune escape mechanism, the amplification of oncogene MYC was widespread in C1, which could further inhibit immune surveillance by increasing the expression of CD47 and PD-L1 (51). Topper and colleagues had demonstrated that depletion of MYC could reversed immune evasion in mouse, which in turn achieved the purpose of treating non-small cell lung cancer, corresponding clinical trial is still ongoing (52). In addition, an intervention study indicated that higher methylation levels of SCD1 were related to weight loss in subject, which was consistent with the lower BMI of C1 (53). The unique SMGs of C2 such as ARID1A, ACVR2A, and CDKN2A were closely associated with chromatin remodeling, which could inhibit the ferroptosis process by altering lipid metabolic genes (54, 55). This suggested that we can target chromatin remodeling to develop drugs for C2. Notably, C2 is more sensitive to the multi-kinase inhibitor sorafenib, which might be attributed to its significant copy number alterations in cell cycle-related kinases such as CDK3, CDK8, and MAPK11 (56). Overall, the specific genomic variation landscape of the two subtypes not only might lead to the formation of heterogeneous ferroptosis subtypes, but also partially contributed to the underlying mechanism of their sensitivity to different drugs. In addition, these results also point the directions for drug development and clinical treatment of HCC patients.

Finally, we developed and validated a prognosis signature termed FRRS in three independent cohorts. The high FRRS predominantly associated with poor prognosis. FRRS demonstrated a favorable performance in predicting the prognosis, and was an independent prognosis factor in HCC. Taking into account the close link between FRRS and TME cells, we further explored the potential significance in predicting immunotherapy response and it turned out FRRS also achieved a high accuracy. In addition, the accuracy of FRRS was superior to seven prevalent indicators including TMB, TIDE, MSI score, Merck18, IFGN, CD8, and CD274 in predicting immunotherapy response, which hinted FRRS was a promising marker for selecting patients who might be sensitive to immunotherapy.

Nevertheless, the study also had several limitations. First, owing to the lack of data, our study only considered the interpatient heterogeneity and did not take into account the intratumoral heterogeneity. Second, although we had applied some algorithms to assess the two subtypes in predicting the sensitivity of sorafenib and immunotherapy, prospective cohort studies and clinical data are still need.

In summary, our work identified and validated two heterogeneous ferroptosis subtypes. The two subtypes also exhibited heterogeneity in functional status, immune escape mechanisms, genome-driven events, and clinical outcomes. In addition, we developed a scoring system termed FRRS, which was a reliable prognosis and immunotherapy signature. These results facilitated the understood of ferroptosis as well as clinical management and precise therapy of HCC.

## Data Availability Statement

The datasets presented in this study can be found in online repositories. The names of the repository/repositories and accession number(s) can be found in the article/**Supplementary Material**.

## Author Contributions

XH and YS designed the research. ZL and LW performed data acquisition and data analysis. LL and TL assisted with data acquisition and data analysis. ZL, LW, and DJ wrote the manuscript. All authors contributed to the article and approved the submitted version.

## Conflict of Interest

The authors declare that the research was conducted in the absence of any commercial or financial relationships that could be construed as a potential conflict of interest.
