# Supplementary material for: The Identification and Validation of Two Heterogenous Subtypes and a Risk Signature Based on Ferroptosis in Hepatocellular Carcinoma
Source: Front Oncol. 2021 Mar 2;11:619242. doi: 10.3389/fonc.2021.619242 (PMC7961086; doi:10.3389/fonc.2021.619242)
Supplement: Supplementary file 1 [file DataSheet_1.docx]

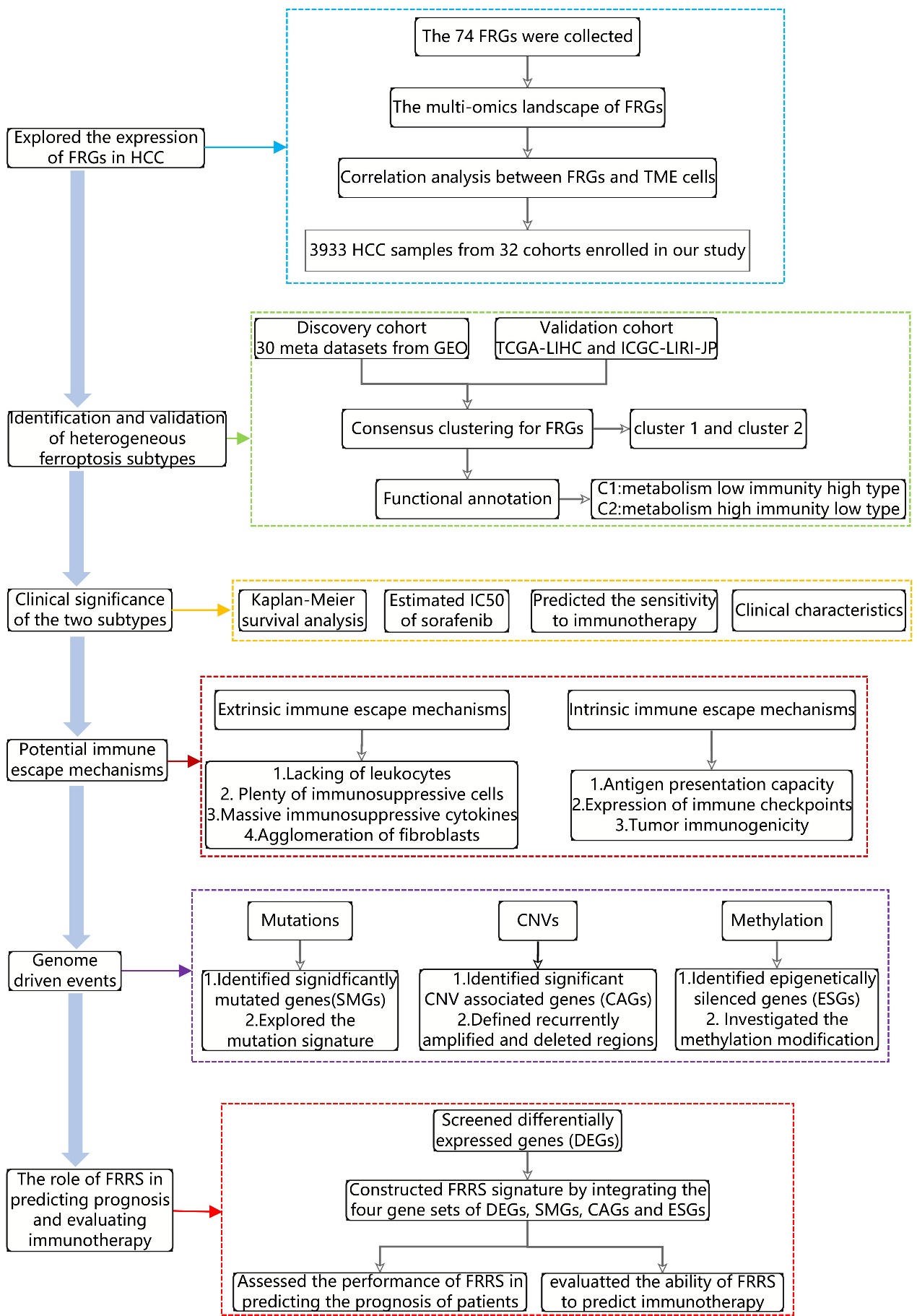


**Supplementary Figure 1** The workflow of our research.


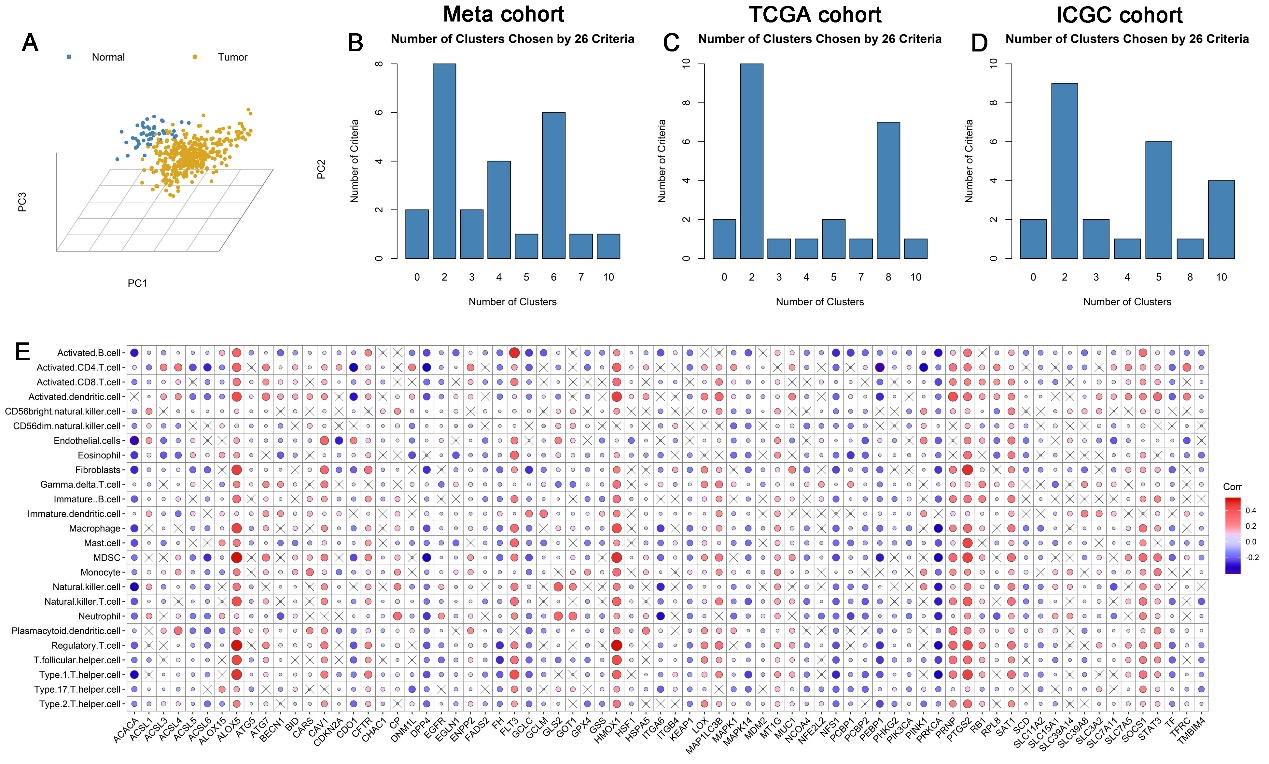


**Supplementary Figure 2** (**A**) Two-dimensional principle component plot by the expression of 74 FRGs in tumor and normal samples. The orange dots represented C1, and blue dots represented C2. (**B-D**) The NbClust algorithm indicated the number of clusters chosen by 26 criteria in the discovery (**B**), TCGA (**C**) and ICGC (**D**) cohorts. (**E**) Spearman correlation analysis between the 74 FRGs expression and TME cells.


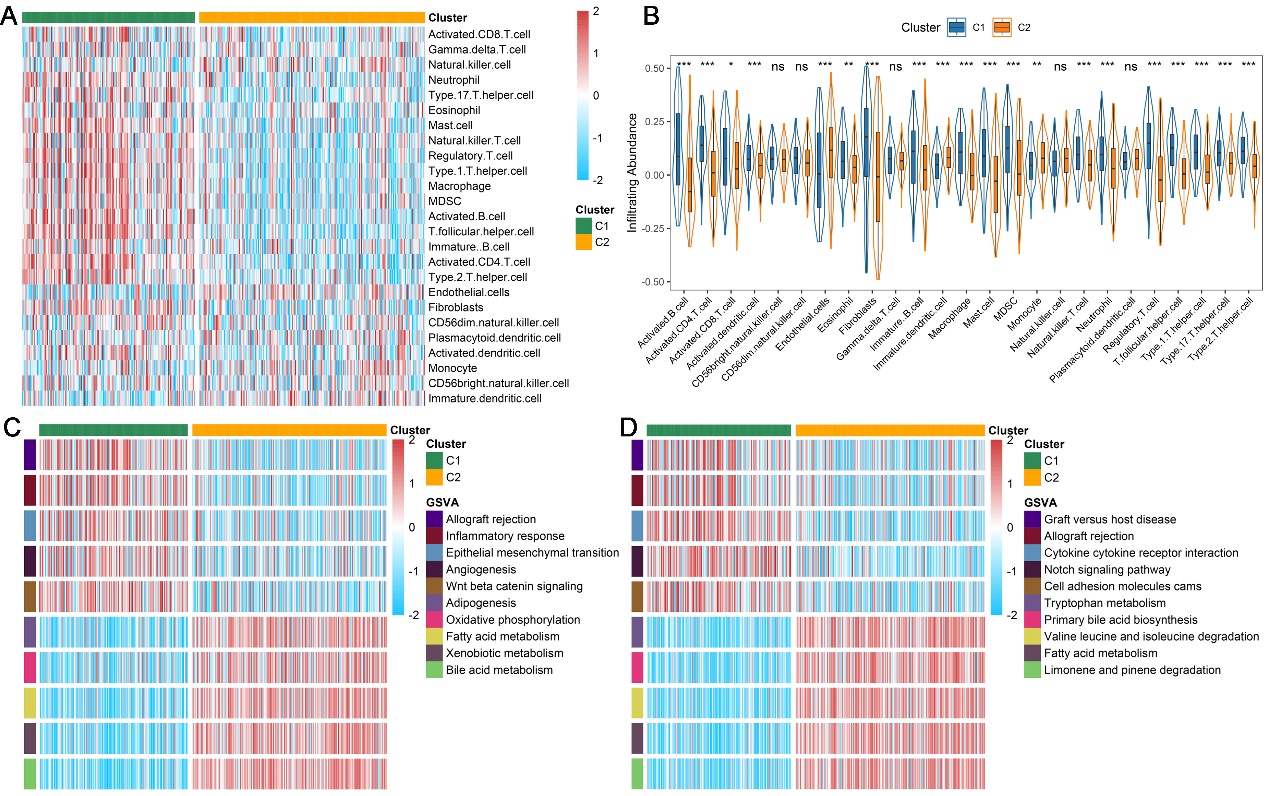


**Supplementary Figure 3** (**A**) The heatmap of immune cells in the two subtypes. (**B**) The relative infiltration abundance of TME cells between the two subtypes. The asterisks represented the statistical p value (^ns^P > 0.05; *P < 0.05; **P < 0.01; ***P < 0.001). (**C, D**) GSVA enrichment analysis revealed activated Hallmark (**C**) and KEGG (**D**) pathways of the two subtypes.


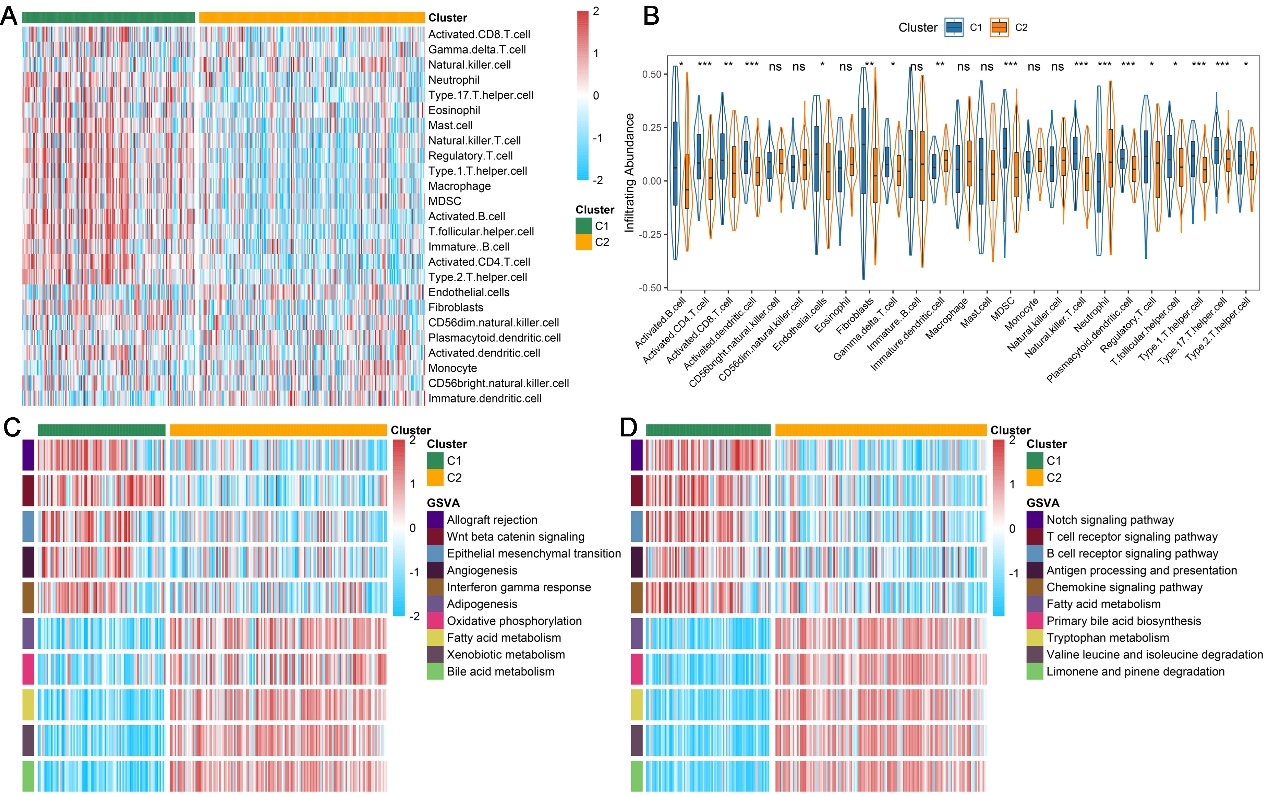


**Supplementary Figure** **4** (**A**) The heatmap of TME cells in the two subtypes. (**B**) The relative infiltration abundance of TME cells between the two subtypes. The asterisks represented the statistical p value (^ns^P > 0.05; *P < 0.05; **P < 0.01; ***P < 0.001). (**C, D**) GSVA enrichment analysis revealed activated Hallmark (**C**) and KEGG (**D**) pathways of the two subtypes.


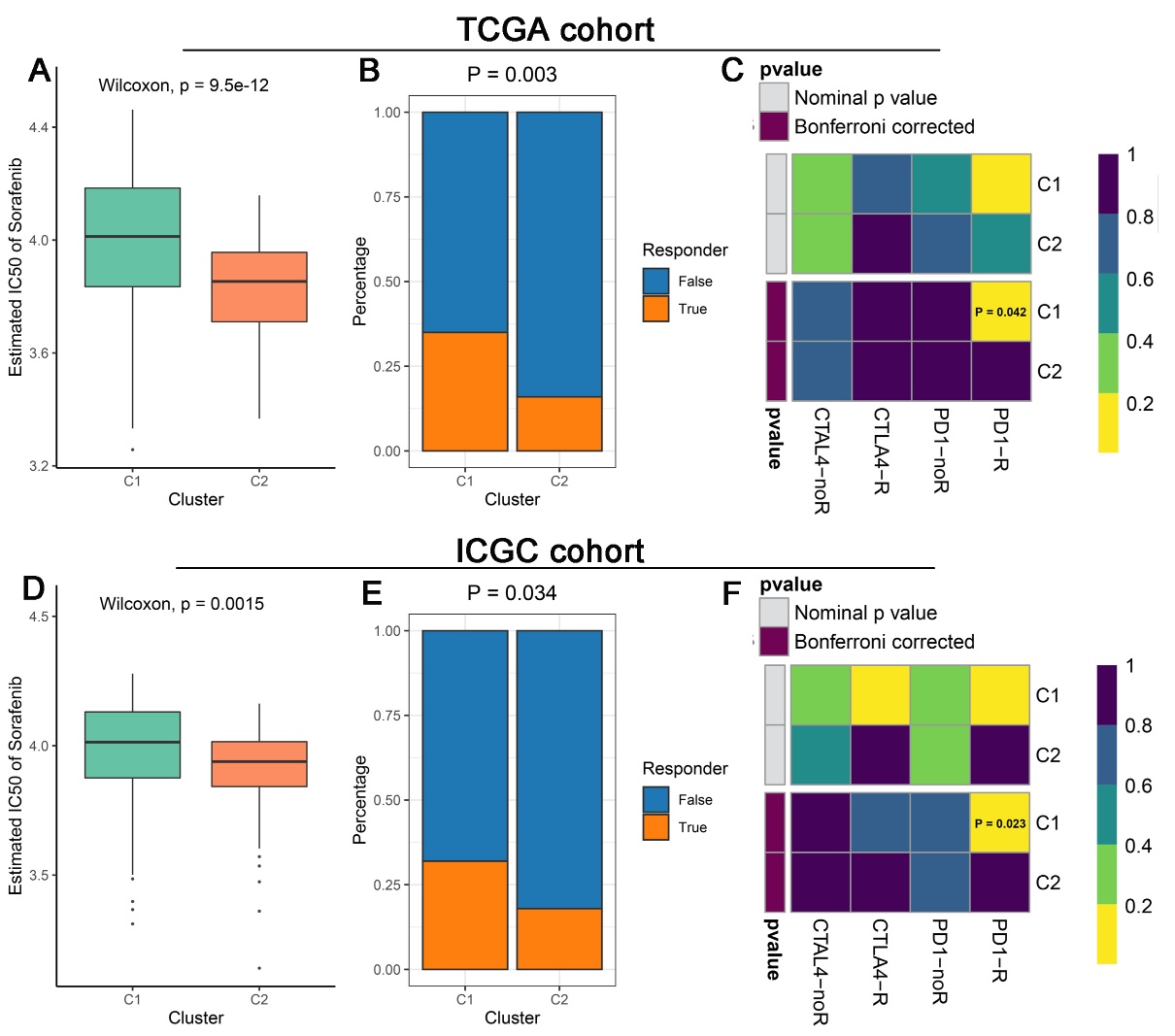


**Supplementary Figure** **5** (**A-C**) The estimated IC50 of sorafenib between the two subtypes (**A**), the TIDE algorithm was used to predict the sensitivity of the two subtypes to immunotherapy (**B**), and Submap analysis of the two subtypes and 47 pretreated patients with comprehensive immunotherapy annotations (**C**) in the TCGA cohort. (**D-F**) The estimated IC50 of sorafenib between the two subtypes (**D**), the TIDE algorithm was used to predict the sensitivity of the two subtypes to immunotherapy (**E**), and Submap analysis of the two subtypes and 47 pretreated patients with comprehensive immunotherapy annotations (**F**) in the ICGC cohort. For Submap analysis, a smaller p-value implied a more similarity of paired expression profiles.


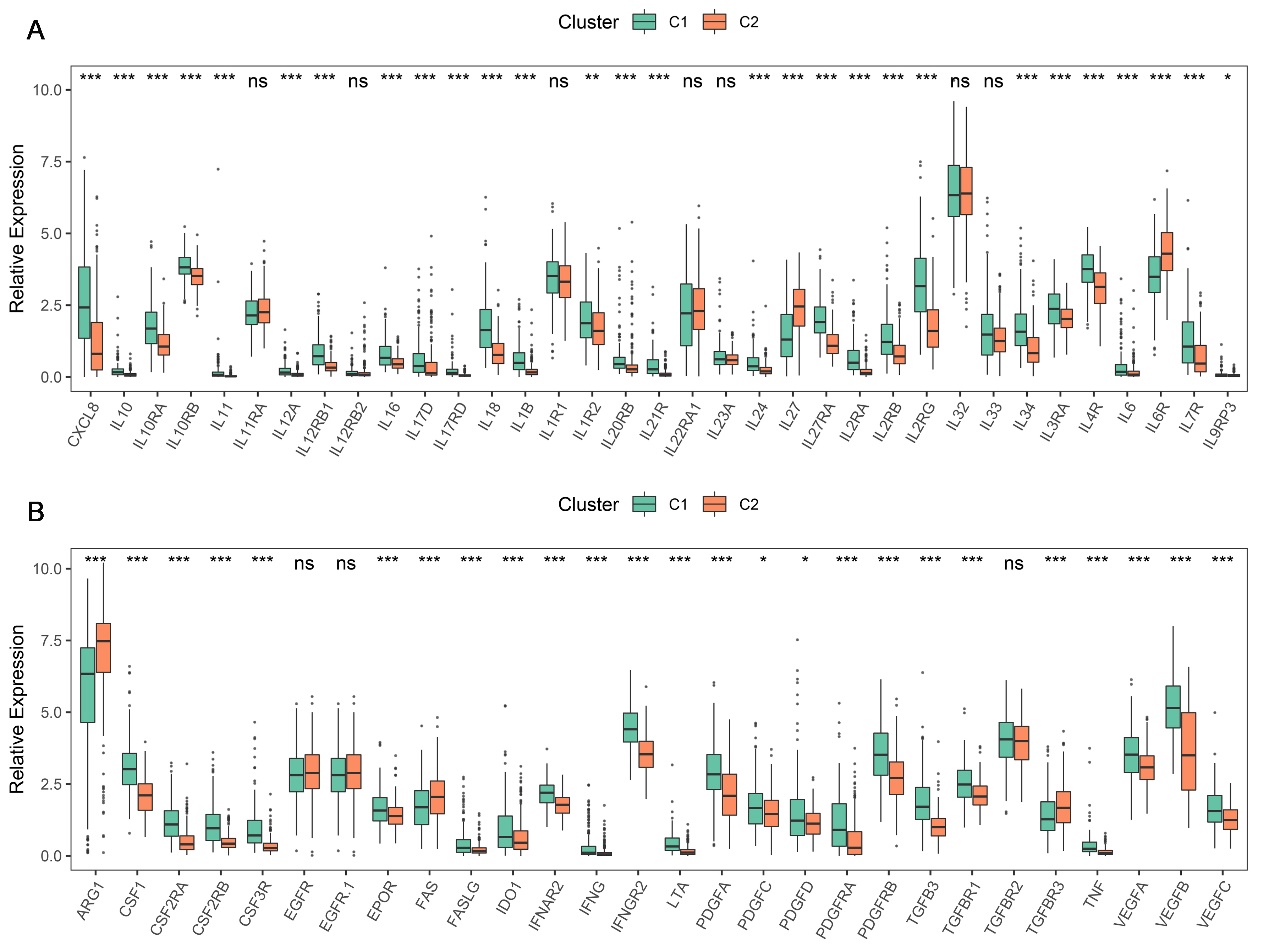


**Supplementary Figure 6** (**A**) The relative expression of interleukins and their receptors in the two subtypes. (**B**) The relative expression of interferons, other crucial cytokines and their receptors in the two subtypes. The asterisks represented the statistical p value (^ns^P > 0.05; *P < 0.05; **P < 0.01; ***P < 0.001).


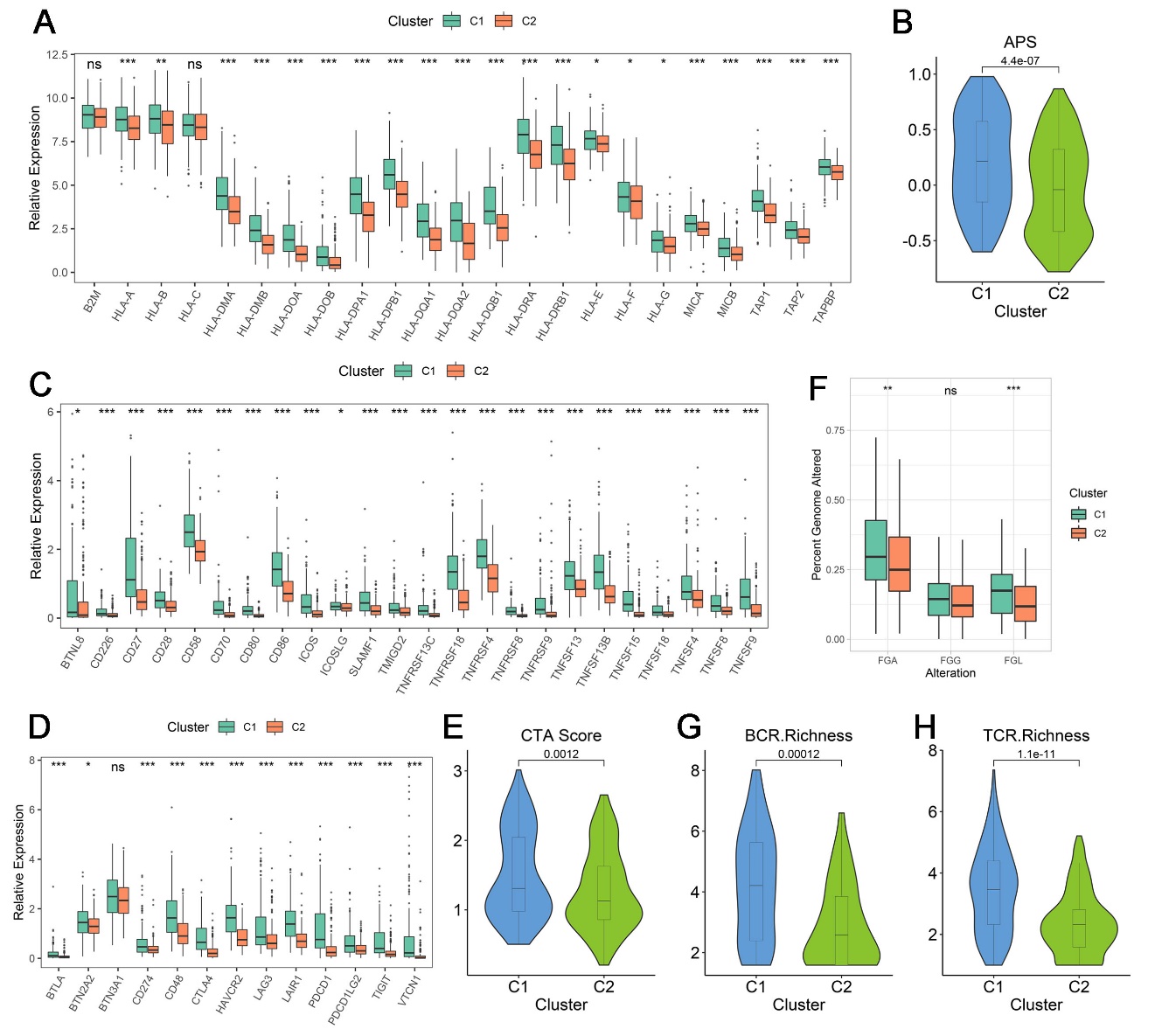


**Supplementary Figure 7** (**A**) The relative expression of MHC molecules in the two subtypes. (**B**) Comparison of APS between the two subtypes. (**C, D**) The relative expression of co-stimulatory and co-inhibitory molecules in two subtypes. (**E**) Fraction of the FGA (Fraction Genome Altered), FGG (Fraction Genome Gained) and FGL (Fraction Genome Lost) between the two subtypes. **F** Comparison of CTA score between the two subtypes. (**G, H**) TCR (**G**) and BCR (**H**) diversity scores of the two subtypes obtained by Richness. The asterisks represented the statistical p value (^ns^P > 0.05; *P < 0.05; **P < 0.01; ***P < 0.001).


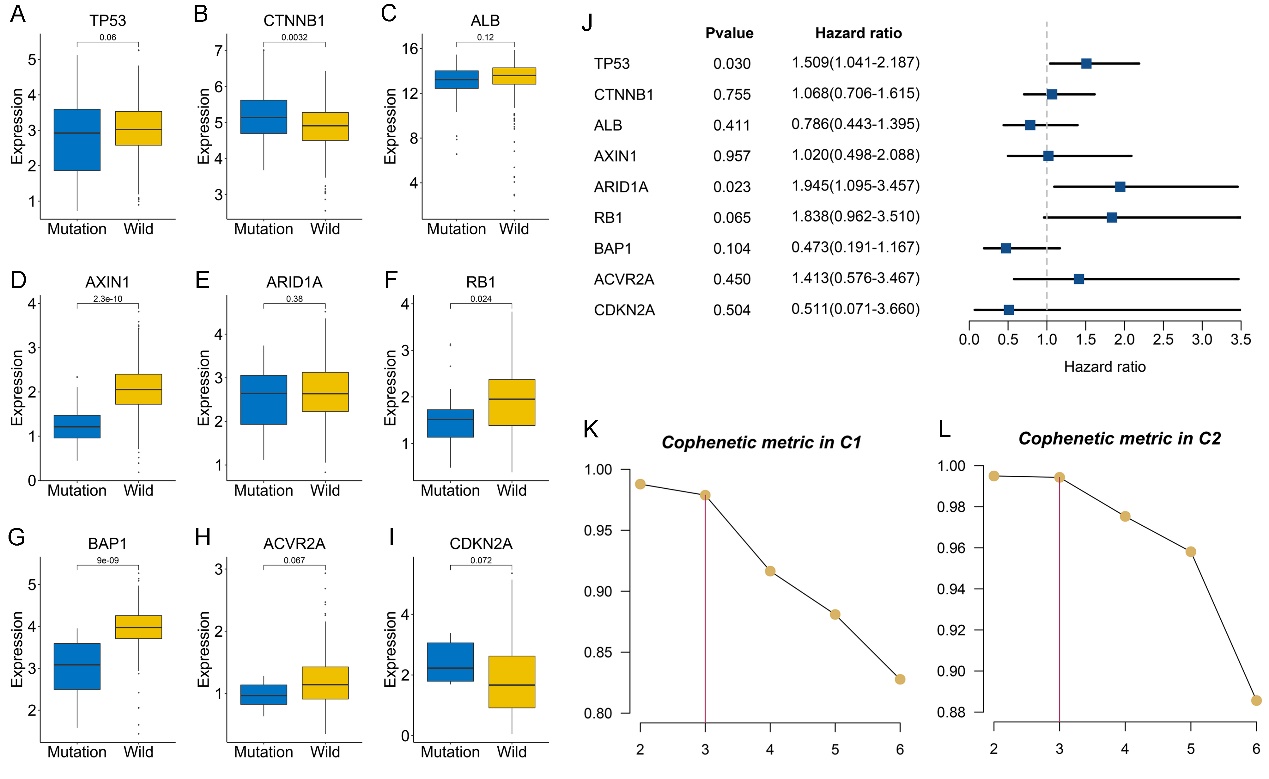


**Supplementary Figure 8** (**A-I**) Expression levels of nine SMGs in wild type and mutant groups. (**J**) Univariate Cox regression analysis of nine SMGs in the TCGA cohort. (**K, L**) The optimal number of C1 (**K**) and C2 (**L**) for mutation signatures identified by non-negative matrix factorization (NMF).


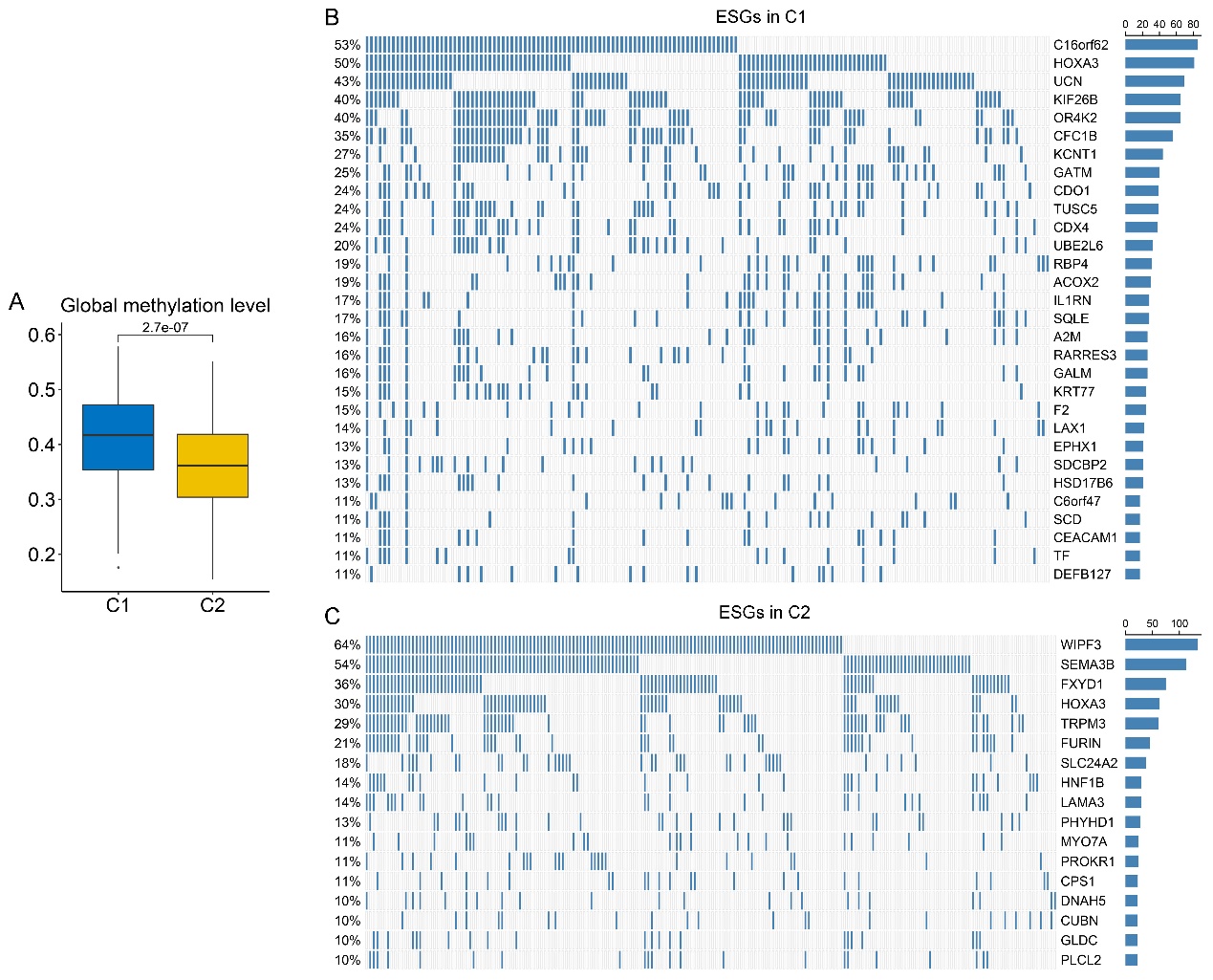


**Supplementary Figure 9 (A)** The distribution of GML in two subtypes. (**B, C**) The ESGs in C1 (**B**) and C2 (**C**). Each column represented individual patients. The number on the left showed the proportion of samples in the whole that this gene was identified as an ESG. The right barplot indicated the total number of samples identified as an ESG in each gene.


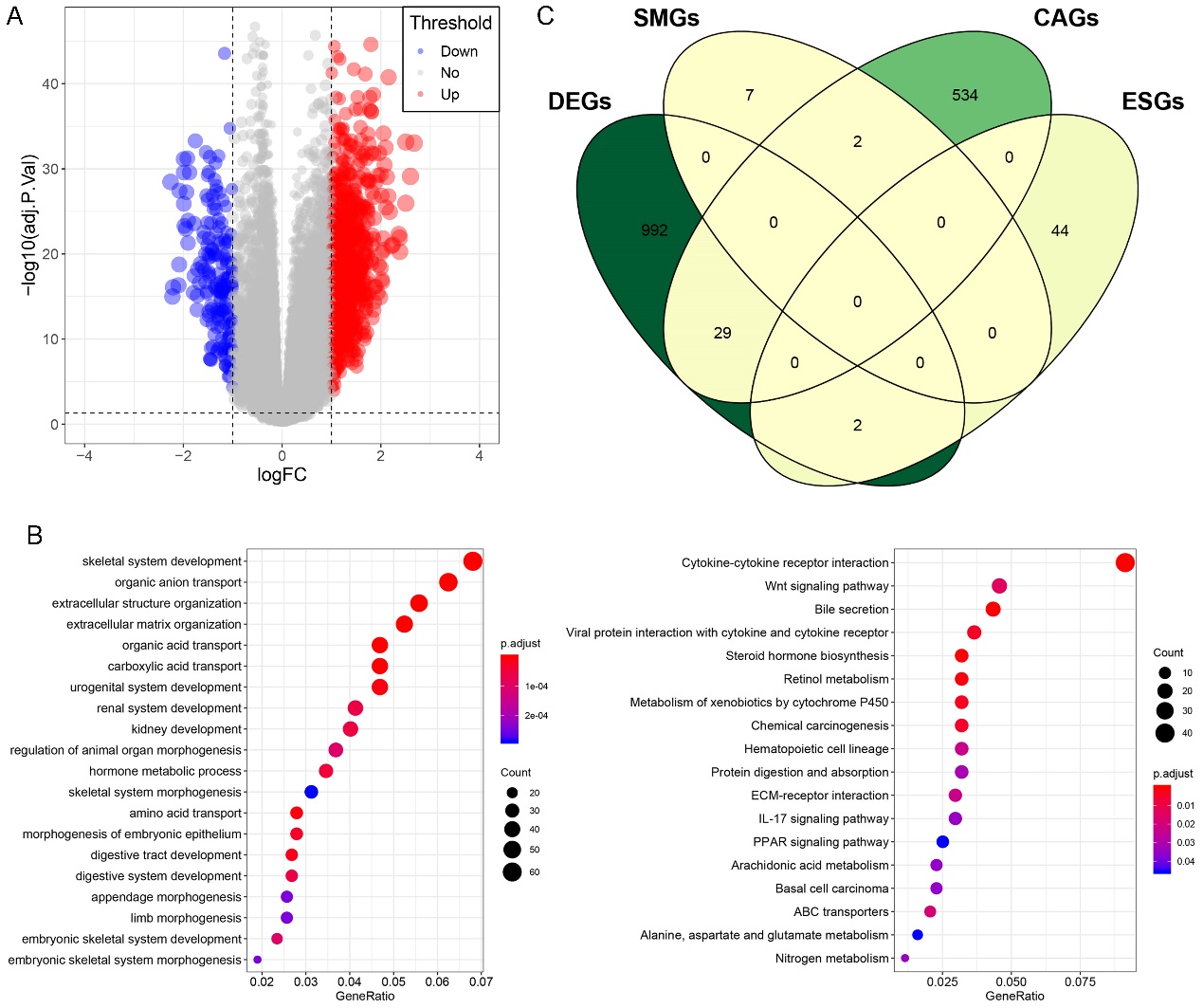


**Supplementary Figure 10** (**A**) Identification the DEGs between the two subtypes. Red dots represented up-regulated genes, blue dots represented down-regulated genes and grey dots represented genes with no significance. (**B**) GO and KEGG enrichment analysis of subtype-relevant DEGs. The size of the circle represented the number of genes enriched on this pathway. The color represented the corrected P value. (**C**) The Venn diagram showed the number of overlapping genes among the four gene sets including DEGs, SMGs, CAGs and ESGs.


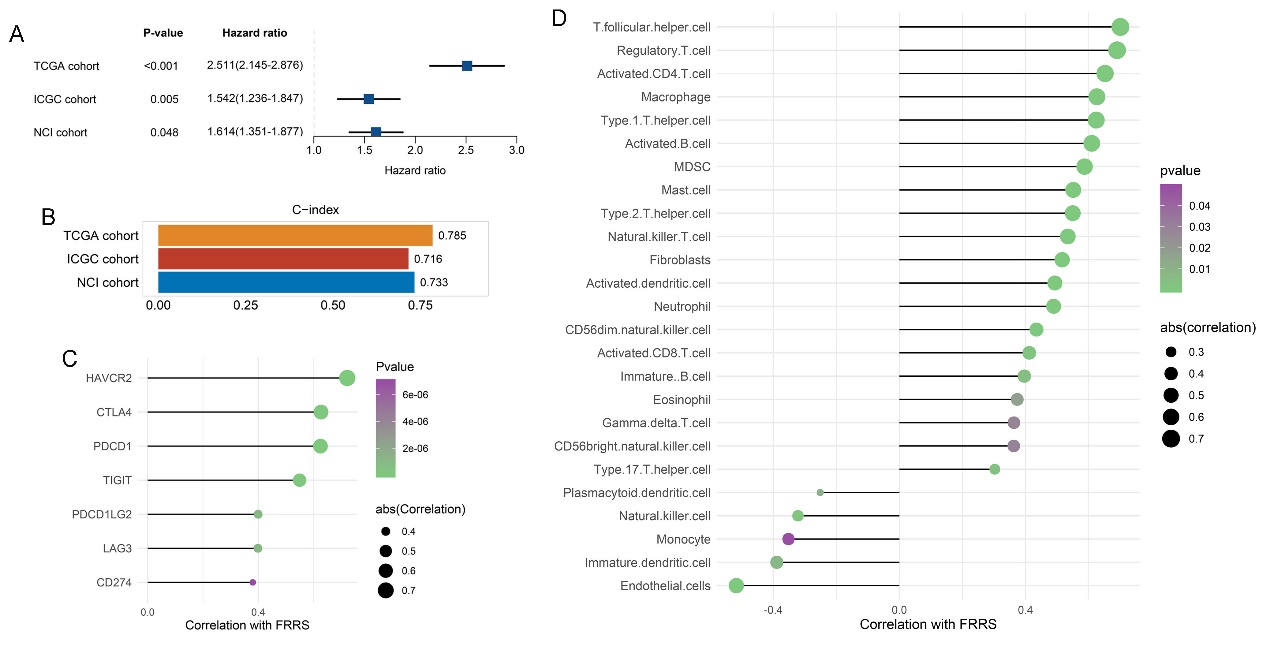


**Supplementary Figure 11** (**A**) Univariate Cox regression analysis of the FRRS in TCGA, ICGC and NCI cohorts. (**B**) C-index of the FRRS signature in TCGA, ICGC and NCI cohorts. (**C**) Spearman correlation analysis between FRRS and the expression of ICP molecules. The size of the circle represented the strength of the relationship, and the color represented the size of the P value. **(D)** Spearman correlation analysis between FRRS and the infiltration abundance of TME cells. The size of the circle represented the strength of the relationship, and the color represented the size of the P value.
